# Preoperative Neutrophil-to-Lymphocyte Ratio, Platelet-to-Lymphocyte Ratio, and CEA as the Potential Prognostic Biomarkers for Colorectal Cancer

**DOI:** 10.1155/2022/3109165

**Published:** 2022-01-04

**Authors:** Fu Ming-Sheng, Du Mei-Ling, Cai Xun-Quan, Hu Yuan-Xin, Zhang Wei-Jie, Pan Qin-Cong

**Affiliations:** Department of Gastroenterology, Shanghai Fifth People's Hospital Fudan University, No. 801, Heqing Road, Minhang District, Shanghai 200240, China

## Abstract

**Background:**

This study was to evaluate the prognostic value of the preoperative neutrophil-to-lymphocyte ratio (NLR), platelet-to-lymphocyte ratio (PLR), and carcinoembryonic antigen (CEA) in colorectal cancer (CRC) patients and to identify the potential and easily accessible prognostic biomarkers for CRC.

**Methods:**

We retrospectively reviewed altogether the records of 330 CRC patients according to inclusion criteria. The clinical characteristics include age at diagnosis, body mass index (BMI), preoperative CEA level, neutrophil , lymphocyte, and platelet count, tumor primary site and size, clinical pathological TNM stage, and survival status were recorded through the review of medical records. The overall survival (OS) was calculated using the Kaplan–Meier method. The Cox proportional hazards model was used for the univariate and multivariate analysis to evaluate the prognostic factors of CRC.

**Results:**

A total of 330 patients were finally included in the current study. The mean follow-up duration was 32.8 ± 19.1 months (range, 0.1–67.7). Compared with the median OS, preoperative high NLR, PLR, and CEA, and low BMI had lower median OS. The NLR and PLR value rise indicates lower median OS in stage I-II CRC; however, the NLR value and CEA level rise indicates lower median OS in stage III-IV CRC. Preoperative high NLR, PLR, and CEA level and low BMI have poorer OS by univariate analysis. By multivariate analysis, the age, sex, N, M stage, and BMI demonstrated independently influence the OS of CRC. NLR was an independent predictor of stage I-II CRC, and the CEA level was an independent predictor of stage III-IV CRC.

**Conclusions:**

Our results show that preoperative high NLR, PLR, CEA, and low BMI had poorer OS, NLR was an independent predictor of stage I-II CRC, and the CEA level was an independent predictor of stage III-IV CRC.

## 1. Introduction

Colorectal cancer (CRC) is the third most common cancer worldwide, and the incidence of CRC in China is rising continuously in recent years; however, most of the patients were still diagnosed in the advanced stage leading to unsatisfactory prognosis for them [1]. The prognoses of CRC are mainly influenced by the completeness of surgical resection and the pathological stage [2–4]. Thus, it is urgent for us to identify the effective potential prognostic biomarkers for the survival improvement of CRC patients.

As we all know, systemic inflammatory response plays a vital role as a leading cause of the neoplastic process, and it was actively engaged in the genesis and propagation of various cancers [5, 6]. We know that systemic inflammation can be reflected by the parameters of peripheral blood including white blood cells, neutrophils, lymphocytes, and platelets. The neutrophil-to-lymphocyte ratio (NLR) and the platelet-to-lymphocyte ratio (PLR) have been confirmed to be the prognosis indicators for many malignancies such as biliary tract cancer and gastric cancer [7, 8]. Systemic inflammation has been linked to poor prognosis of colorectal cancer [9]. Recently, preoperative systemic inflammation indexes are considered as promising prognostic predictors which have easy accessibility and convenient application [10]. Elevated NLR and PLR have been associated with poor survival of colorectal cancer [11]. Carcinoembryonic antigen (CEA) is regarded as the common serological biomarker for the detection and monitoring of CRC but has insufficient sensitivity and specificity for prognostic [12].

Therefore, the aim of this study was to evaluate the prognostic values of preoperative NLR and PLR and CEA levels in CRC patients. In order to identify the potential and easily accessible prognostic biomarkers for CRC.

## 2. Materials and Methods

### 2.1. Patients

The retrospective analysis was conducted in patients with histologically confirmed colorectal adenocarcinoma who underwent surgical resection in the Department of Gastrointestinal Surgery at Shanghai Fifth People's Hospital, Fudan University between January 1, 2015, and December 31, 2017. The exclusion criteria were (1) clinical confirmation of infectious disease or other diseases that caused systemic inflammation before surgery, (2) patients diagnosed with previous or concurrent malignancies, (3) patients with hematologic disorders, (4) patients with cirrhosis, and (5) patients who received steroid therapy. At last, 330 patients were enrolled in this study, and informed consent was obtained from all patients. This study was approved by the ethics committee of Shanghai Fifth People's Hospital, Fudan University.

### 2.2. Blood Samples and Reference Values

Blood samples were drawn from venous blood within 1 week before the date of surgery by a nurse. The blood samples are tested for complete blood count and the CEA value. The reference range of CEA value is 0.0–4.7 ng/mL, the reference range is (2.0–7.0) × 10^9^/L for neutrophils count, (100–300) × 10^9^/L for platelets count, and (0.80–4.0) × 10^9^/L for lymphocytes count. The NLR and PLR were calculated by dividing the absolute number of neutrophils or platelets by the absolute number of lymphocytes, respectively.

### 2.3. Evaluation of Clinical Characteristics

The clinical characteristics of all colorectal cancer patients, including age at diagnosis, sex, body mass index (BMI), preoperative CEA level, neutrophil, lymphocyte, and platelet count, NLR, PLR, tumor primary site (rectum, colon), tumor size (diameter <5 cm, ≥5 cm), clinical pathological stage (stage I-II, stage III-IV), TNM stage (AJCC, version7), and survival status (alive/died), were recorded through the review of medical records.

The overall survival (OS) time was measured from the date of surgery to the date of death from any cause or most recent follow-up. The survival and follow-up data were obtained by collecting outpatient clinical records or by directly contacting the patient or their relatives through a phone call from January 1, 2015, to June 30, 2020.

### 2.4. Statistical Analysis

The optimal cut-off values of preoperative NLR, PLR, BMI, and CEA were determined by the receiver operating characteristic (ROC) curve. Survival analysis was computed using the Kaplan–Meier method and compared by the log-rank test. The Cox proportional hazards model with a 95% confidence interval was used for the univariate analysis and multivariate analysis to assess the effect of patient characteristics and other significant prognostic factors. All statistical tests were two-sided, and associations were considered statistically significant at a nominal level of 0.05 (*P* < 0.05). Statistical analysis was performed using the SPSS software for windows (version 25.0).

## 3. Results

### 3.1. Baseline Clinical Characteristics of CRC Patients

A total of 330 patients were finally included in the current study, including 198 (60.0%) males and 132 (40.0%) females. The mean age was 71.7 ± 12.3 years (range, 32–99). The mean BMI was 21.73 ± 1.91 (range, 17.5–31.4). There was no statistical difference between the NLR (*P*=0.382) and PLR (*P*=0.232) in the high and low BMI groups. A total of 201 patients (60.9%) had colon cancer and the remaining 129 patients (39.1%) had rectal cancer. The evaluation of TNM stages revealed that the clinical pathological diagnoses were 163 patients of stage I-II and 167 patients of stage III-IV. The mean follow-up duration was 32.8 ± 19.1 months (range, 0.1–67.7).

### 3.2. NLR, PLR, BMI, and CEA Cut-Off Value

The ROC curve could calculate the sensitivity and specificity levels of NLR, PLR, BMI, and CEA as predictors of CRC survival. The optimal cut-off value of NLR was calculated as 3.03 with the areas under the curve (AUC) = 0.578 (*P*=0.015), a sensitivity of 56.9%, a specificity of 61.3%, and the PLR was 149.7 with AUC = 0.584 (*P*=0.009), a sensitivity of 63.2%, a specificity of 51.6%, and the BMI was 21.7 with AUC = 0.709 (*P* < 0.001), a sensitivity of 67.4%, a specificity of 68.8%, and the CEA was 13.4 with AUC = 0.573(*P*=0.022), a sensitivity of 35.4%, a specificity of 81.2%, by the AUC with the Youden index. All the patients were divided into high and low groups according to the NLR, PLR, BMI, and CEA cut-off values.

### 3.3. Kaplan–Meier Survival Analysis

**Figure 1:**
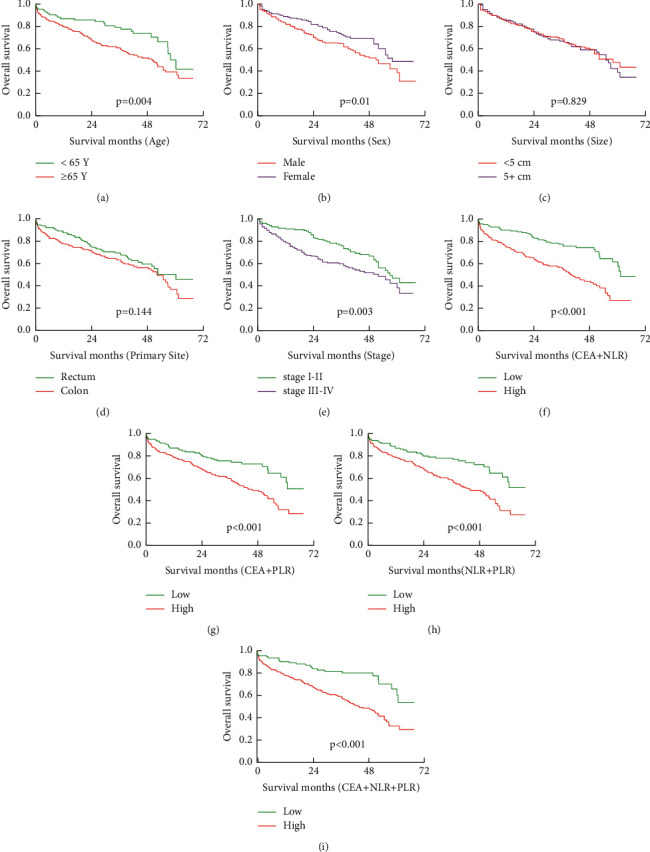
Kaplan–Meier estimates of overall survival (OS) according to the main clinical variables status:(a) according to age status (*P*=0.004), (b) according to sex status (*P*=0.01), (c) according to tumor size status (*P*=0.829), (d) according to primary site status (*P*=0.144), (e) according to stage status (*P*=0.0036), (f) according to CEA + NLR (*P* < 0.001), (g) according to CEA + PLR (*P* < 0.001), (h) according to NLR + PLR (*P* < 0.001), and (i) according to CEA + NLR + PLR (*P* < 0.001).

**Figure 2:**
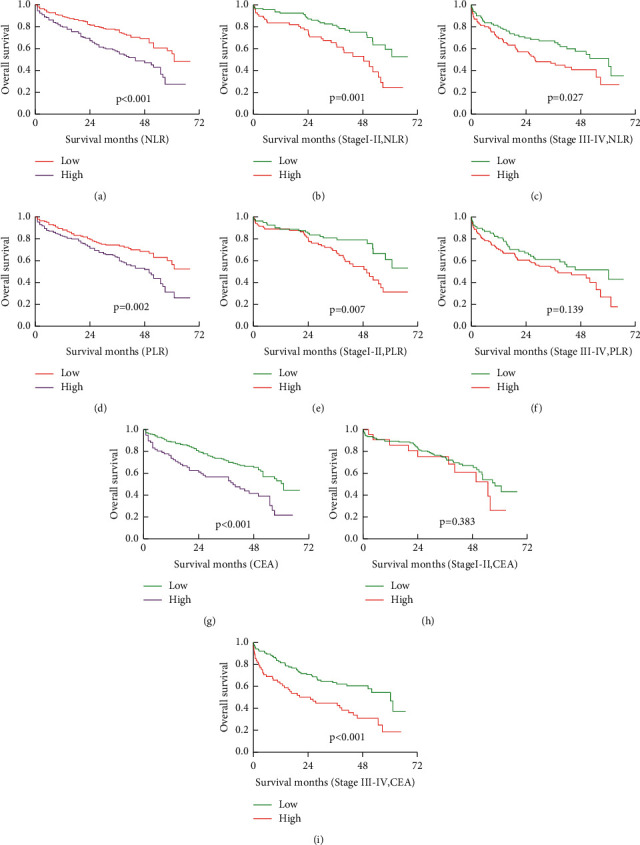
Kaplan–Meier estimates of overall survival (OS) according to the NLR, PLR, and CEA.:(a) according to NLR (*P* < 0.001), (b) according to NLR in stage I-II (*P*=0.001), (c) according to NLR in stage III-IV (*P*=0.027), (d) according to PLR (*P*=0.002), (e) according to PLR in stage I-II (*P*=0.007), (f) according to PLR in stage III-IV (*P*=0.139), (g) according to CEA (*P* < 0.001), (h) according to CEA in stage I-II (*P*=0.383), (i) according to NLR in stage III-IV (*P* < 0.001).

The Kaplan–Meier estimates of overall survival according to main clinical variables status are shown in Tables 1–3 and Figures 1 and 2.  Table 1 shows that the average survival month of colorectal cancer is significantly reduced in the high group of NLR, PLR, CEA, and low BMI group. From Figure 1, we found that the prognosis is poor with over 65 years old, male, stage III-IV, and in the high group of a combination of NLR, PLR, and CEA or the combination of all three. From Table 2 and Figure 2, we found that the NLR value rise in early and advanced colorectal cancer indicates a poor prognosis, and the PLR value rise in early colorectal cancer indicates a poor prognosis. On the contrary, the CEA level elevated in stage III-IV colorectal cancer indicates a poor prognosis. From Table 3, we found that the NLR and CEA elevated in rectum and colon cancer indicates a poor prognosis, and the PLR value rise only in colon cancer indicates a poor prognosis.

### 3.4. Univariate and Multivariate Analysis

For analysis of all variables in prognostic factors, Tables 4–6 show the results of univariate and multivariate analysis of various parameters with OS evaluated in our study.

Our results showed that the CRC patients with preoperative high NLR, PLR, and CEA levels, and the low BMI group have poorer OS in the univariate analysis. The group of over 65 years of age and the group of male patients had a poor prognosis as shown in Table 4. However, there were no significant associations with OS in the primary site, tumor size, and T-stage groups with univariate analysis (Table 4).

Multivariate analysis was performed to assess the independent predictors for survival. As shown in Table 5, the risk of death in the high NLR group was 1.38 times that of the low NLR group (*P*=0.112), the high PLR group was 1.28 times that of the low PLR group (*P*=0.226), the high BMI group was 0.26 times that of the low BMI group (*P* < 0.001), and the high CEA group was 1.24 times that of the low CEA group (*P*=0.268). The results show that age, sex, N, M stage, and BMI demonstrated independently the influence OS of CRC. In addition, when we conducted the study by stage, we found that NLR was an independent predictor of stage I-II colorectal cancer prognosis, and the CEA level was an independent predictor of stage III-IV colorectal cancer prognosis (Table 6).

## 4. Discussion

Various studies confirmed that inflammatory response plays an important role in the progression of tumor microenvironment, some changes of inflammatory cells might be a predictor for prognosis, and changes of immune cellular components in peripheral venous blood could reflect tumor inflammation status for predicting survival prognosis [13]. Since complete blood count and CEA level are routinely measured as part of the preoperative work-up of patients undergoing surgery, their possible prognostic value could be very relevant in clinical practice.

The roles of lymphocytes in tumor immune surveillance and immunoediting have been widely studied [14]. Lymphocytes can eliminate tumor cells through the cytotoxic effects [15]. On the contrary, neutrophils and monocytes may contribute to tumor progression. Neutrophils release prostaglandin E2 (PGE2) to amplify inflammation and create a tumor microenvironment, which can promote colon tumorigenesis, suppress activities of natural killer cells, and increase the exudation of tumor cells through the secretion of interleukin-1*β*(IL-1*β*) and matrix metalloproteinases (MMP) [16]. Besides, neutrophils can release neutrophil extracellular traps to promote hepatic metastasis of CRC by trapping tumor cells [17]. Therefore, NLR is an integrated indicator for the pro-tumor effect of neutrophils and the antitumor immunity of lymphocytes. It has been demonstrated that high levels of platelets are capable of promoting cancer progression by increasing angiogenesis through the production of the vascular endothelial growth factor (VEGF), overexpression of which has been associated with disease progression and metastasis in patients with CRC [18]. In addition, platelets could secrete cellular growth factors such as platelet-derived growth factor, vascular endothelial growth factor, transforming growth factor-beta, and platelet factor 4 and then stimulate tumor angiogenesis and growth [19]. Therefore, elevated preoperative platelet counts probably signify an organic microenvironment conducive to tumor growth. PLR can reflect the balance between the cancer promotion capacity of platelet and the anti-tumor immunity of lymphocytes. CEA is mainly used for assistant diagnosis of malignant tumors, determining prognosis, and monitoring curative effect and recurrence of tumors, and it is most effective when patients have high preoperative serum CEA levels [20]. However, sensitivity is far from being sufficient [21]. Plasma CEA level is not consistently elevated in CRC and may be undetectable or present at only low levels with a poorly differentiated tumor [22].

Our results showed that the patients of preoperative high NLR, PLR, and CEA level had poorer OS by the Kaplan–Meier analysis. Similarly, the results showed that the OS of the high group was significantly reduced in the CEA combined with NLR or PLR, and the NLR combined with PLR. In addition, the OS of CRC patients who were male, over 65 years of age, had lymph node invasion, and had distant metastasis was poor. Furthermore, through the clinical pathological stage grouping analysis, in the stage I-II, the mean OS of high NLR and PLR was significantly reduced, but there is no statistical difference in OS in the CEA high and low groups. While, in the stage III-IV, the mean OS of high NLR, CEA was significantly reduced, and there was no statistical difference in OS between PLR high and low groups. When we grouped by primary site and analyzed, we found that the mean OS of high NLR, CEA was significantly reduced, and there was no statistical difference in OS between PLR high and low groups in the rectum. In the colon, the mean OS of high NLR, PLR, CEA was significantly reduced. The univariate Cox proportional hazards model analysis result was similar to the Kaplan–Meier analysis result. The multivariate Cox proportional hazards model analysis result showed that the age over 65, being male, N1–N2, M1 stage, and low BMI were independent risk factors for poor prognosis of CRC, without stratified analysis, NLR, PLR, and CEA level cannot be determined as independent risk factors for the prognosis of CRC. However, it was interesting that we found that high NLR value was independent risk factors for poor prognosis in Stage I-II group patients with CRC by stage conducted stratified, while in stage III-IV CRC patients, only a high CEA level was independent risk factor for poor prognosis. Our results showed that NLR to be predictive of outcome in CRC patients with stage I-II but not stage III-IV. We speculate that it may be related to the early inflammation and immune of CRC. Similarly, CEA is an independent predictor for advanced CRC (stage III-IV) but not stage I-II, which may be related to the release of higher levels of CEA in advanced CRC. Li [23] reported 5336 patients with CRC; the result showed that H-NLR was an independent prognostic factor for OS at multivariate analysis. Haram's study concluded that preoperative NLR > 5 was associated with poorer OS in patients with CRC, they conducted a systematic review to assess the prognostic role of NLR in metastatic and non-metastatic CRC [24]. Zhou [25] reported postoperative inflammation indexes such as neutrophil and monocyte to lymphocyte ratio (NMLR), systemic immune inflammation index (SII), and C-reaction protein (CRP) to albumin (ALB) ratio (CAR) are promising prognostic predictors of CRC patients. Malietzis [26] reported 506 patients with non-metastatic CRC who did not receive adjuvant chemotherapy; the result showed that an independent prognostic role of H-NLR (>3) was not identified. Furthermore, because of the difference in treatment and prognosis of the colon and rectal cancer, most of the studies included colon as well as rectal cancer, therefore producing results that may be biased [27]. The PLR has been demonstrated as a prognostic factor in gastric cancer [28] and esophageal carcinoma [29]. Previous research has demonstrated that the platelet addition to tumor cells can impede natural killer cell-mediated recognition and elimination of tumor cells, which may prime the tumor cells for metastasis [30]. In addition, NLR and PLR are related to non-neoplastic diseases, such as alcoholic liver cirrhosis (ALC) and nonalcoholic fatty liver disease (NAFLD). They are closely related to indirect and direct markers of liver fibrosis. Moreover, the NLR and PLR seem to correlate with a clinical progression of liver cirrhosis [31]. Milovanovic's [32] study demonstrates that patients with NAFLD have a significant increase in the values of platelet indices(PCT), mean platelet volume (MPV), and platelet distribution width (PDW) when compared to the healthy controls. El-Gazzar's [33] report showed that the NLR and PLR increased in stable COPD patients and further increased during exacerbation that can predict in-hospital mortality. Gasparyan [34] reported that the PLR and NLR have high predictive value in rheumatic diseases with predominantly neutrophilic inflammation. Li's [35] study has shown that the NLR and PLR are the independent factors that affect the disease activity of rheumatoid arthritis patients and can better evaluate the disease activity and efficacy of rheumatoid arthritis. Our results show that the low BMI in CRC has a poor prognosis. Jaspan [36] reported that obese and underweight BMI are associated with increased CRC-specific and overall mortality compared to that of normal BMI. Long-term prognosis was similar for patients with obese and underweight BMI. The previous report showed that the mGPS and CEA accurately predict OS in patients with liver metastasis from CRC [37].

The main limitation of our study relates to its retrospective nature and the limited sample size. However, our study also has many strengths. First, all the patients have complete follow-up data after surgery. Second, we find some potential prognostic markers through stratified analysis.

## 5. Conclusion

In summary, whether it is a univariate or multivariate analysis, preoperative low BMI is a risk factor for poor prognosis of CRC. The preoperative high CEA level is an independent risk factor for poor prognosis in stage III-IV CRC patients. Besides, high NLR significantly affects survival in stage I-II CRC patients; it is an independent risk factor for poor prognosis in stage I-II CRC patients. In order to confirm our observations and identify the effective clinical survival value of NLR, PLR, and CEA, a higher number of cases are required for further studies in the future.

## Figures and Tables

**Table 1:** Survival analysis using the Kaplan–Meier and compared by the log-rank test.

Characteristics		*N*	Mean OS (months)	*X* ^2^	*P* value
Age	<65 Y	87	51.5	8.3	0.004
	≥65 Y	243	41.7		

Sex	Male	198	41	6.7	0.01
	Female	132	48.6		

Primary site	Rectum	129	47.4	2.1	0.144
	Colon	201	42.3		

Tumor size	<5 cm	205	44.1	0.1	0.829
	5+ cm	125	45		

T stage	T0–T2	68	48.7	2.3	0.138
	T3–T4	262	43.2		

N stage	N0	185	48.5	8.9	0.003
	N1–N3	145	38.7		

M stage	M0	275	46.9	17.9	<0.001
	M1	55	30.7		

Stage	I-II	163	49.2	9.1	0.003
	III-IV	167	39.2		

NLR	<3.03 (low)	176	49.7	16.6	<0.001
	≥3.03 (high)	154	37.4		

PLR	<149.73 (low)	148	49.2	9.7	0.002
	≥149.73 (high)	182	40.2		

CEA (ng/mL)	<13.4 (low)	244	47.9	17.9	<0.001
	≥13.4 (high)	86	33.3		

CEA + NLR	Low	141	52.6	24.4	<0.001
	High	189	37.4		

CEA + PLR	Low	117	51.6	13.4	<0.001
	High	213	40.4		

NLR + PLR	Low	116	52	15.5	<0.001
	High	214	40		

CEA + NLR + PLR	Low	94	54.7	19.3	<0.001
	High	236	40.2		

BMI	<21.7 (low)	155	35.1	35.5	<0.001
	≥21.7 (high)	175	52.3		

NLR, neutrophil-to-lymphocyte ratio; PLR, platelet-to-lymphocyte ratio; CEA, carcinoembryonic antigen; BMI, body mass index.

**Table 2:** Kaplan–Meier survival analysis and log-rank test compared in the clinical pathological stage.

Characteristics		Stage I-II				Stage III-IV			
		*N*	Mean OS (months)	*X* ^2^	*P* value	*N*	Mean OS (months)	*X* ^2^	*P* value
NLR	Low	94	54.2	10.7	0.001	82	43.9	4.9	0.027
	High	69	41.4			85	34		

PLR	Low	80	54.1	7.4	0.007	68	42.9	2.2	0.139
	High	83	44.9			99	35.5		

CEA (ng/mL)	Low	140	49.8	0.7	0.383	104	44.9	12.4	<0.001
	High	23	43.9			63	29.5		

CEA + NLR	Low	83	54.4	8.6	0.003	58	49.6	12.1	0.001
	High	80	42.9			109	33.2		

CEA + PLR	Low	71	53.7	5.2	0.023	46	47.4	5.6	0.018
	High	92	45.9			121	35.4		

NLR + PLR	Low	64	56.2	9.9	0.002	52	46.2	4.9	0.027
	High	99	44.7			115	35.3		

CEA + NLR + PLR	Low	58	55.6	7.2	0.007	36	52.4	9.5	0.002
	High	105	45.6			131	35		

**Table 3:** Kaplan–Meier survival analysis and log-rank test compared in the primary site.

Characteristics		Rectum				Colon			
		*N*	Mean OS (months)	*X* ^2^	*P* value	*N*	Mean OS (months)	*X* ^2^	*P* value
NLR	Low	80	51.4	6.5	0.011	96	48.1	8.5	0.004
	High	49	39.5			105	36.3		

PLR	Low	71	48.5	1.4	0.234	77	48.9	7.3	0.007
	High	58	44.9			124	37.5		

CEA (ng/mL)	Low	106	49.7	4.9	0.027	138	46.5	11.8	0.001
	High	23	35.4			63	32.1		

CEA + NLR	Low	69	52.4	6.3	0.012	72	52.8	16.9	<0.001
	High	60	40.9			129	35.4		

CEA + PLR	Low	59	50.7	3.6	0.057	58	51.2	8.7	0.003
	High	70	43.8			143	37.9		

NLR + PLR	Low	59	48.4	1.9	0.164	57	53.4	13.6	<0.001
	High	70	44.8			144	37.2		

CEA + NLR + PLR	Low	49	49.8	2.8	0.096	45	57.7	17.6	<0.001
	High	80	44.7			156	37.1		

**Table 4:** Univariate analysis of overall survival by the Cox proportional hazards model.

Characteristics		*N*	HR	95% CI	*P* value
Age	<65 Y	87	REF	1.21–2.82	0.005
	≥65 Y	243	1.84		

Sex	Male	198	REF	0.45–0.90	0.011
	Female	132	0.64		

Primary site	Rectum	129	REF	0.91–1.81	0.149
	Colon	201	1.29		

Tumor size	<5 cm	205	REF	0.69–1.35	0.83
	5+ cm	125	0.96		

T stage	T0–T2	68	REF	0.90–2.14	0.138
	T3–T4	262	1.39		

N stage	N0	185	REF	1.18–2.26	0.003
	N1–N3	145	1.63		

M stage	M0	275	REF	1.52–3.30	<0.001
	M1	55	2.24		

Stage	I-II	163	REF	1.19–2.30	0.003
	III-IV	167	1.65		

NLR	Low	176	REF	1.41–2.73	<0.001
	High	154	1.96		

PLR	Low	148	REF	1.21–2.40	0.002
	High	182	1.70		

CEA (ng/mL)	Low	244	REF	1.46–2.89	<0.001
	High	86	2.05		

CEA + NLR	Low	141	REF	1.67–3.42	<0.001
	High	189	2.39		

CEA + PLR	Low	117	REF	1.36–2.90	<0.001
	High	213	1.99		

NLR + PLR	Low	116	REF	1.44–3.09	<0.001
	High	214	2.11		

CEA + NLR + PLR	Low	94	REF	1.65–3.99	<0.001
	High	236	2.57		

BMI	Low	155	REF	0.26–0.52	<0.001
	High	175	0.36		

**Table 5:** Multivariate analysis of overall survival by the Cox proportional hazards model.

Characteristics		*N*	HR	95% CI	*P* value
Age	<65 Y	87	REF	0.99–2.38	0.053
	≥65 Y	243	1.54		

Sex	Male	198	REF	0.26–0.56	<0.001
	Female	132	0.38		

Primary site	Rectum	129	REF	0.90–1.88	0.158
	Colon	201	1.30		

Tumor size	<5 cm	205	REF	0.62–1.34	0.639
	5+ cm	125	0.91		

T stage	T0–T2	68	REF	0.44–1.23	0.248
	T3–T4	262	0.74		

N stage	N0	185	REF	1.10–2.92	0.02
	N1–N3	145	1.79		

M stage	M0	275	REF	1.05–2.74	0.03
	M1	55	1.70		

Stage	I-II	163	REF	0.49–1.41	0.499
	III-IV	167	0.83		

NLR	Low	176	REF	0.92–2.06	0.112
	High	154	1.38		

PLR	Low	148	REF	0.86–1.91	0.226
	High	182	1.28		

CEA (ng/mL)	Low	244	REF	0.85–1.81	0.268
	High	86	1.24		

BMI	Low	155	REF	0.18–0.39	<0.001
	High	175	0.26		

**Table 6:** Multivariate analysis of overall survival by the clinical pathological stage.

Characteristics		Stage I-II			Stage III-IV		
		HR	95% CI	*P* value	HR	95% CI	*P* value
Age	<65 Y	REF	0.83–3.19	0.156	REF	1.08–3.40	0.027
	≥65 Y	1.63			1.91		

Sex	Male	REF	0.39–1.15	0.141	REF	0.37–1.03	0.064
	Female	0.66			0.62		

Primary site	Rectum	REF	0.68–2.06	0.546	REF	0.64–1.69	0.881
	Colon	1.19			1.04		

Tumor size	<5 cm	REF	0.38–1.33	0.279	REF	0.61–1.69	0.945
	5+ cm	0.71			1.02		

T stage	T0–T2	REF	0.45–1.76	0.744	REF	0.31–1.77	0.499
	T3–T4	0.89			0.74		

N stage	N0	REF	0.45–2.93	0.775	REF	0.73–2.38	0.354
	N1–N3	1.14			1.32		

M stage	M0	REF	1.71–184.99	0.016	REF	0.99–2.81	0.052
	M1	17.79			1.68		

NLR	Low	REF	1.14–3.75	0.017	REF	0.61–1.77	0.891
	High	2.06			1.04		

PLR	Low	REF	0.89–3.14	0.108	REF	0.79–2.25	0.290
	High	1.67			1.33		

CEA (ng/mL)	Low	REF	0.69–2.91	0.342	REF	1.13–2.85	0.013
	High	1.42			1.79		

## Data Availability

The data used to support the findings of this study are included in the article.
